# The metabolic effects of habitual leg shaking: A randomized crossover trial

**DOI:** 10.1111/1753-0407.13556

**Published:** 2024-04-25

**Authors:** Riqiang Bao, Yixiang Hu, Rui Xu, Chong Gao, Yuhan Guo, Yashu Zhu, Shijia Pan, Weiqing Wang

**Affiliations:** ^1^ Department of Endocrine and Metabolic Diseases Shanghai Institute of Endocrine and Metabolic Diseases, Ruijin Hospital, Shanghai Jiaotong University School of Medicine Shanghai China; ^2^ Shanghai National Clinical Research Center for Metabolic Diseases, Key Laboratory for Endocrine and Metabolic Diseases of the National Health Commission of the PR China, Shanghai National Center for Translational Medicine, Ruijin Hospital, Shanghai Jiaotong University School of Medicine Shanghai China; ^3^ National Research Center for Translational Medicine Shanghai China; ^4^ Shanghai Digital Medicine Innovation Center Shanghai China

**Keywords:** carbohydrate oxidation, energy expenditure, leg shaking, metabolic equivalent, sedentary behavior

## Abstract

**Aims:**

The adverse effects of sedentary behavior on obesity and chronic diseases are well established. However, the prevalence of sedentary behavior has increased, with only a minority of individuals meeting the recommended physical activity guidelines. This study aimed to investigate whether habitual leg shaking, a behavior traditionally considered unfavorable, could serve as an effective strategy to improve energy metabolism.

**Materials and Methods:**

A randomized crossover study was conducted, involving 15 participants (mean [SD] age, 25.4 [3.6]; mean [SD] body mass index, 22 [3]; 7 women [46.7%]). The study design involved a randomized sequence of sitting and leg shaking conditions, with each condition lasting for 20 min. Energy expenditure, respiratory rate, oxygen saturation, and other relevant variables were measured during each condition.

**Results:**

Compared to sitting, leg shaking significantly increased total energy expenditure [1.088 kj/min, 95% confidence interval, 0.69–1.487 kj/min], primarily through elevated carbohydrate oxidation. The average metabolic equivalent during leg shaking exhibited a significant increase from 1.5 to 1.8. Leg shaking also raised respiratory rate, minute ventilation, and blood oxygen saturation levels, while having no obvious impact on heart rate or blood pressure. Electromyography data confirmed predominant activation of lower leg muscles and without increased muscle fatigue. Intriguingly, a significant correlation was observed between the increased energy expenditure and both the frequency of leg shaking and the muscle mass of the legs.

**Conclusions:**

Our study provides evidence that habitual leg shaking can boost overall energy expenditure by approximately 16.3%. This simple and feasible approach offers a convenient way to enhance physical activity levels.

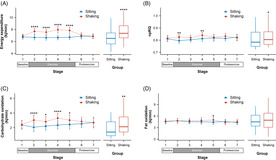

## Graphical abstract

Highlights

- Previous research has established the detrimental effects of sedentary behavior on obesity and chronic diseases. Efforts to mitigate heightened sedentary behavior through measures such as promoting elevated physical activity or incorporating extended standing and exercise during sedentary intervals have faced challenges in implementation.
- Our study confirmed that habitual leg shaking effectively increased energy expenditure by approximately 16.3%, elevated the metabolic equivalent to a nonhealthy level, enhanced carbohydrate oxidation, improved blood oxygen saturation and minute ventilation, while avoiding additional cardiovascular burden.
- Leg shaking offers a simple and feasible approach to enhance physical activity without disrupting daily routines.

## 1 INTRODUCTION

In recent decades, the prevalence of obesity and related chronic disease has surged. In China, 34.3% of adults are overweight, with 16.4% obesity rate; 38.1% have prediabetes and 12.4% have diabetes.^1^, ^2^ This is primarily attributed to imbalances between elevated energy intake and reduced energy expenditure. Notably, numerous epidemiological studies have identified high levels of total sedentary behavior (SB) as an independent factor for obesity, diabetes, cardiovascular disease, and all‐cause mortality.^3^, ^4^, ^5^ SB is defined as any waking behavior expending ≤1.5 metabolic equivalent tasks (METs) while seated, lying, or in a reclined posture.^6^ As sedentary leisure activities and jobs continue to grow,^7^, ^8^, ^9^ particularly in the COVID‐19 era,^10^ individuals, especially in office employees, spend an average of 8–12 h per day sitting.^3^, ^9^, ^11^ Although 60–75 min of daily moderate‐to‐vigorous intensity physical activity can counteract the risk of prolonged sitting.^12^ It is noteworthy that current physical activity guidelines promote a threshold of 150 min of moderate‐to‐vigorous physical activity per week although only very few adults meet these guidelines.^13^, ^14^ Some studies have attempted to address this issue by promoting prolonged standing and walking or incorporating exercise during sedentary periods, implementing these strategies in real‐life settings often presents numerous barriers.^15^, ^16^, ^17^, ^18^, ^19^, ^20^ Hence, finding simple, accessible strategies to boost energy expenditure is vital.

Leg shaking, a common stereotype involving repetitive and rhythmic movements of the legs while seated, occurs in healthy populations apart from individuals with medical conditions such as restless leg syndrome or attention deficit hyperactivity disorder and is often considered undesirable.^21^, ^22^ Some devices and software even aim to detect users' leg shaking to reduce its occurrence.^23^ However, studies have consistently found that over half of the healthy population experiences this behavior.^22^, ^24^ The soleus and the gastrocnemius, the two largest lower leg muscles, are composed mainly of slow oxidative fibers (70% in the soleus and 50% in the gastrocnemius).^25^, ^26^ These fibers, with abundant capillaries, mitochondria, aerobic respiratory enzymes, and myoglobin, can function for extended periods without fatigue and are crucial for maintaining posture, producing isometric contractions, stabilizing bones and joints, and making small movements that happen often but do not require large amounts of energy.^27^

Therefore, we conducted a randomized crossover study to systematically evaluate whether habitual leg shaking, a behavior traditionally considered unfavorable, could serve as a simple, accessible, and effective strategy to improve energy metabolism.

## 2 MATERIALS AND METHODS

### 2.1 Ethical approval

**FIGURE 1:**
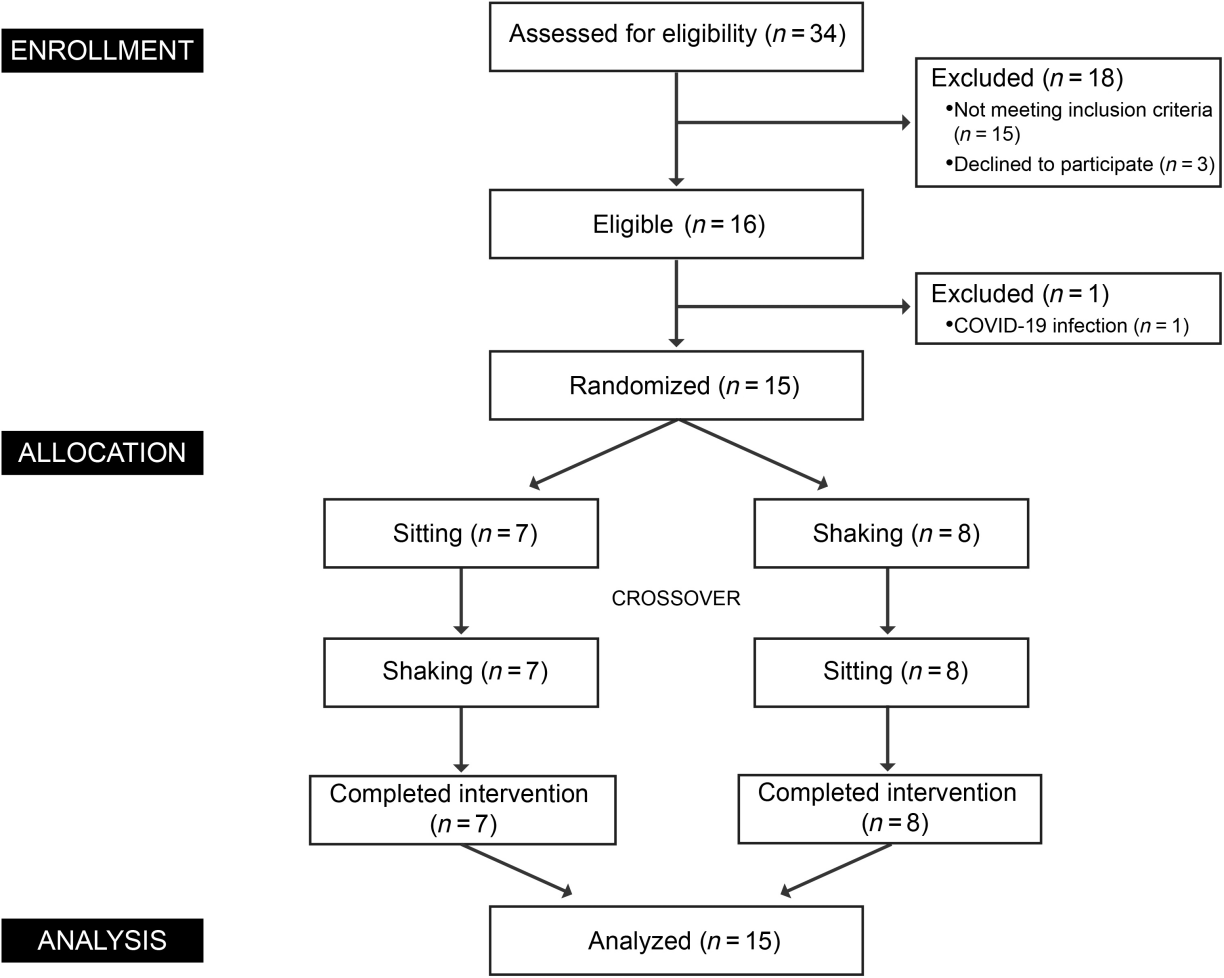
Consolidated Standards of Reporting Trials flow diagram indicating the design of the trial.

The study protocol was approved by the ethics committee of Ruijin Hospital affiliated to Shanghai Jiaotong University. The study was conducted according to the Declaration of Helsinki. Clinical trial registered as “The effect of leg shaking on human energy metabolism” at chictr.org.cn as ChiCTR2300069965. A Consolidated Standards of Reporting Trials flow diagram outlining the study protocol is displayed in Figure 1.

### 2.2 Participants

Sixteen healthy participants were recruited from 34 subjects who were interested in this study. Finally, 15 participants completed this study. Eligibility criteria included 18 ≤ age ≤ 40, 18.5 ≤ body mass index (BMI) ≤25 kg/m^2^, stable weight, a healthy lifestyle, absence of caffeine or alcohol consumption habit, and nonsmoking. We excluded participants with shift work or jet lag within a month and during the experiment, medication use over the past 3 months, and diseases with abnormal clinical manifestations that need to be excluded. Participants were required to abstain from strenuous physical exercises, alcohol, and caffeine 48 h before the study. All participants provided written informed consent prior to participation in the trial.

### 2.3 Study design

Participants maintained a fasting state overnight before arriving at the laboratory to mitigate the influence of the thermic effect of food. The study commenced at ~8:00 a.m. and the study environment was strictly controlled: the temperature was maintained at 25°C (±0.1°C), humidity at 50% (±1%), and the intensity of light and ambient noise was kept consistent. Each participant underwent an initial 20‐min resting metabolic rate (RMR) measurement. Subsequently, based on randomization, they engaged in either a sitting or leg shaking group for a 20‐min duration, followed by a within‐subject crossover. There was a 20‐min resting period between intervention. During the leg shaking intervention, participants were instructed to oscillate their left leg vertically at a frequency consistent with their habitual pattern. Throughout the study, participants maintained a relaxed sitting posture, minimized movements, and refrained from using electronic devices.

### 2.4 Metabolic measurements

Energy expenditure and substrates oxidation were assessed using the K5 wearable metabolic system (COSMED, Rome, Italy).^28^, ^29^ The calorimeter uses a galvanic fuel cell and a nondispersive infrared sensor to analyze the level of O_2_ and CO_2_ in the inhaled and exhaled air. A high‐performance turbine flowmeter was employed to measure flow rate. After a 30‐minute warmup, flowmeter and gas analyzer were calibrated following instructions. Two‐point gas calibration was completed using the reference gas (5.0% CO_2_, 15.0% O_2_, 79% N_2_) and ambient air. A CO_2_ scrubber was used to zero the CO_2_ analyzer. Flowmeter calibration was performed by connecting the turbine to a calibrated 3‐L syringe. The calorimeter collected breath‐by‐breath CO_2_ and O_2_ production and consumption, respectively, and energy expenditure was calculated using the Weir equation.^30^

### 2.5 Physiological parameters measurements

Noninvasive blood pressure, 3‐lead electrocardiography, and blood oxygen saturation (SpO_2_) were continuously monitored by a Cardiac Telemetry System (WEP‐5204C, Nihon Kohden Co., Tokyo, Japan). Systolic and diastolic blood pressure were measured every 10 min during the study. Heart rate and SpO_2_ were measured every second during the study.

### 2.6 Anthropometric measurements

Participant' body weight, height, and body composition were measured using a bioelectrical impedance analysis scale (Inbody 770, Inbody Co. Ltd., Seoul, Korea), an ultrasonic instrument (HNH‐318, OMRON Corporation, Kyoto, Japan), and a dual‐energy X‐ray absorptiometry scanner (GE Lunar iDXA, GE Healthcare, Madison, WI, USA). Body parameters were measured by 3Dscanner (Body‐Scan V5, JDSCAN Co. Ltd., Shanghai, China).

### 2.7 Electromyography measurements

Surface electromyography (EMG) signals were recorded using Ultium EMG (Noraxon USA Inc., Scottsdale, AZ, USA) for gluteus maximus, gluteus medius, rectus femoris, biceps femoris, tibialis anterior, medial gastrocnemius, and soleus on the left leg throughout the study. The attachment sites of the electrode on the skin were shaved and cleaned with alcohol. Disposable self‐adhesive Ag/AgCl surface electrodes were then attached at locations 2 cm apart over each muscle belly and parallel to the orientation of the muscle fibers. The EMG signals were processed and analyzed with MR 3.18 software (Noraxon Inc). Raw EMG was collected at 2000 Hz and band‐pass filtered (Lancosh FIR) between 10 and 500 Hz. The EMG data were rectified and further smoothed using the root mean square with a moving window of 50 ms. During the intervention, the 20‐min period was divided into four stages, and 60 s of raw EMG data were selected for analysis in each stage. Each peak of EMG activity represented the activation of the corresponding muscle during a leg shaking cycle. A total of *n* = 14 EMG measurements were made as an equipment malfunction voided one subject's dataset.

### 2.8 Statistical analysis

Based on the previous research demonstrating that single muscle group activation led to a significant increase in energy expenditure (4.69 ± 0.53 kJ/min) compared to the control group (4.27 ± 0.53 kJ/min),^31^ we calculated that a sample size of 15 participants per group would provide this study 80% power to detect the difference of energy expenditure between sitting and leg shaking conditions at the alpha value of 0.05. This calculation was performed using G*Power (version 3.1.9.2; G*Power Software, Düsseldorf, Germany).

Participants were subjected to a randomized crossover intervention, with random allocation sequence generation based on a random‐number table. Stratification by gender was also implemented in the randomization process. The characteristics of the participants were summarized using descriptive statistics and raw mean (± SD) or median (interquartile range) were used to describe normally and nonnormally distributed quantitative variables, respectively. Data for each group during intervention are presented as raw mean ± SEM. The Shapiro–Wilk test was employed to assess the data distribution. For parameters with normal Gaussian distribution were analyzed using a two‐tailed paired Student's *t* test. Treatment effect of intervention between sitting and leg shaking conditions at different stages were reported as least squares mean ± SEM. Differences between groups were estimated using a linear mixed‐effects model with treatment group, sequence, and stage were treated as fixed effects; the participants served as the random effects. Where significant, post‐hoc tests were performed using Bonferroni corrections. Repeated measures analysis of variance was employed to test the differences of median power frequency (MPF) during leg shaking. To assess the correlation between variables, the Pearson correlation coefficients were calculated. *p* < .05 was set as the significance threshold. All statistical analyses were conducted using R version 4.06.

## 3 RESULTS

### 3.1 Participants

The number of participants who were screened, enrolled, and completed the study was shown in Figure 1. From March 30 to April 30, 2023, 16 participants were enrolled and randomized to either sitting or leg shaking intervention, with crossover later. Ultimately, 15 participants (mean [SD] age, 25.4 [3.6]; mean [SD] BMI, 22 [3]; 7 women [46.7%]) completed the study. Baseline characteristics were shown in Table 1. No significant differences were found in biochemical or physiological profiles between groups.

**TABLE 1:** Basal characteristics of participants.

Characteristics	Sitting‐first group	Shaking‐first group	*p* value^a^
No. (%) of participants (*n* = 15)	7 (46.7)	8 (53.3)	
Gender			
Women (%)	4 (57.1)	3 (37.5)	
Men (%)	3 (42.9)	5 (62.5)	
Age (years), mean (SD)	24.86 (2.85)	25.88 (4.36)	.598
Height (cm), mean (SD)	165.21 (6.51)	170.56 (5.88)	.122
Weight (kg), mean (SD)	58.63 (10.99)	66.14 (11.85)	.225
BMI (kg/m^2^), mean (SD)	21.33 (2.67)	22.64 (3.29)	.411
DXA			
Fat (%), mean (SD)	28.59 (6.12)	26.69 (4.92)	.525
FFM (kg), mean (SD)	41.34 (7.96)	48.07 (8.7)	.142
Muscle of left leg (kg), mean (SD)	6.42 (1.86)	7.78 (1.69)	.168
Neck circumference (cm), mean (SD)	40.17 (10.81)	38.35 (1.96)	.728
Waist circumference (cm), mean (SD)	77.09 (5.45)	84.69 (8.72)	.114
Hip circumference (cm), mean (SD)	93.78 (5.56)	96.7 (5.84)	.419
Calf circumference (cm), mean (SD)	36.45 (1.86)	35.83 (2.99)	.682
RMR (kj/min), mean (SD)	6.69 (1.75)	7.12 (1.3)	.604
Heart rate (beats/min), mean (SD)	86.17 (10.53)	85.51 (11.85)	.925
Respiratory rate (breaths/min), mean (SD)	20.16 (3.21)	20.05 (2.78)	.951
SpO_2_ (%), mean (SD)	97.81 (0.37)	97.9 (0.19)	.599
Systolic blood pressure (mm Hg), mean (SD)	71.6 (14.64)	71.8 (5.47)	.978
Diastolic blood pressure (mm Hg), mean (SD)	104.77 (13.52)	109 (7.36)	.56

Abbreviations: BMI, body mass index; DXA, dual‐energy X‐ray absorptiometry; FFM, fat‐free mass; RMR, resting metabolic rate; SpO_2_, peripheral oxygen saturation.

^a^ Differences between two groups were tested by independent samples *t* test.

### 3.2 Metabolic parameters

**FIGURE 2:**
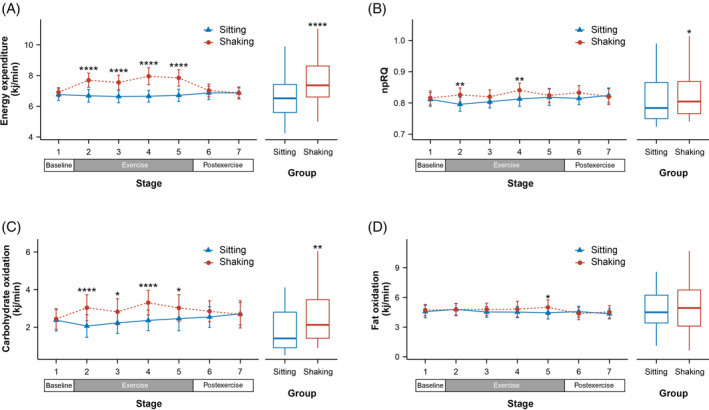
Effects of leg shaking on metabolic parameters. Energy expenditure (A), npRQ (B), carbohydrate oxidation (C), and fat oxidation (D) between sitting and leg shaking group. Left panel: Values at different stages presented as LSM ± SEM, *p* values obtained from linear mixed modeling. Right panel: Mean values during intervention presented as mean ± SEM, *p* values obtained from paired Student's *t* test. *p < .05, **p < .01, ****p < .0001. LSM, least squares mean; npRQ, nonprotein respiratory quotient.

The data included a 5‐min baseline, a 20‐min exercise, and a 10‐min postexercise period. It was divided into seven stages with 5‐min intervals. No differences were observed during the baseline period (Figure 2). However, changes occurred immediately after leg shaking began. In comparison to the sitting group, leg shaking significantly increased energy expenditure throughout the exercise period (all *p* ≤ .0001). The average increase in energy expenditure was 1.088 kj/min (95% confidence interval [CI], 0.69–1.487 kj/min), ~16.3% higher than sitting. The average METs also showed a significant elevation from 1.5 to 1.8 (delta, 95% CI, 0.158–0.318) during the leg shaking period. Leg shaking slightly increased npRQ at stage 2 and 5 (all *p* ≤ .007) with an average increase of 0.016 (95% CI, 0.003–0.029). Correspondingly, a significant increase in carbohydrate oxidation was observed during the leg shaking period, reaching 0.736 kj/min (95% CI, 0.268–1.204 kj/min). Fat oxidation exhibited a subtle increase in the final stage of leg shaking (*p* = .013). No differences were found in the postexercise period.

### 3.3 Physiological parameters

**FIGURE 3:**
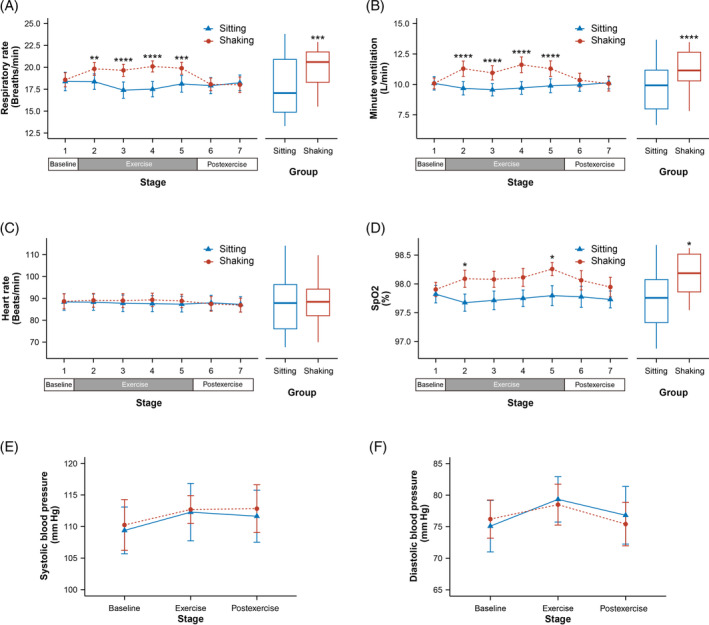
Effects of leg shaking on physiological parameters. Respiratory rate (A), minute ventilation (B), heart rate (C), and SpO_2_ (D), systolic blood pressure (E), and diastolic blood pressure (F) between sitting and leg shaking group. Left panel: Values at different stages presented as LSM ± SEM, *p* values obtained from linear mixed modeling. Right panel: Mean values during intervention presented as mean ± SEM, *p* values obtained from paired Student's *t* test. *p < .05, **p < .01, ***p < .001, ****p < .0001. LSM, least squares mean; SpO_2_, blood oxygen saturation.

As shown in Figure 3, compared to the sitting group, leg shaking significantly increased the respiratory rate (all *p* ≤ .006), with an average increase of 2.061 breaths/min (95% CI, 1.128–2.995 breaths/min). Minute ventilation (VE) also exhibited a significant increase (all *p* ≤ .0001) and the mean VE increased 1.578 L/min (95% CI, 1.011–2.146 L/min). No significant changes were observed in heart rate and blood pressure. However, SpO_2_ showed a significant increase in stage 2 and 5 (all *p* ≤ .035), and a trend to increase in Stage 3 and 4. On average, SpO_2_ was higher by 0.331% (95% CI, 0.07–0.592%) during leg shaking.

### 3.4 Muscle EMG analysis

**FIGURE 4:**
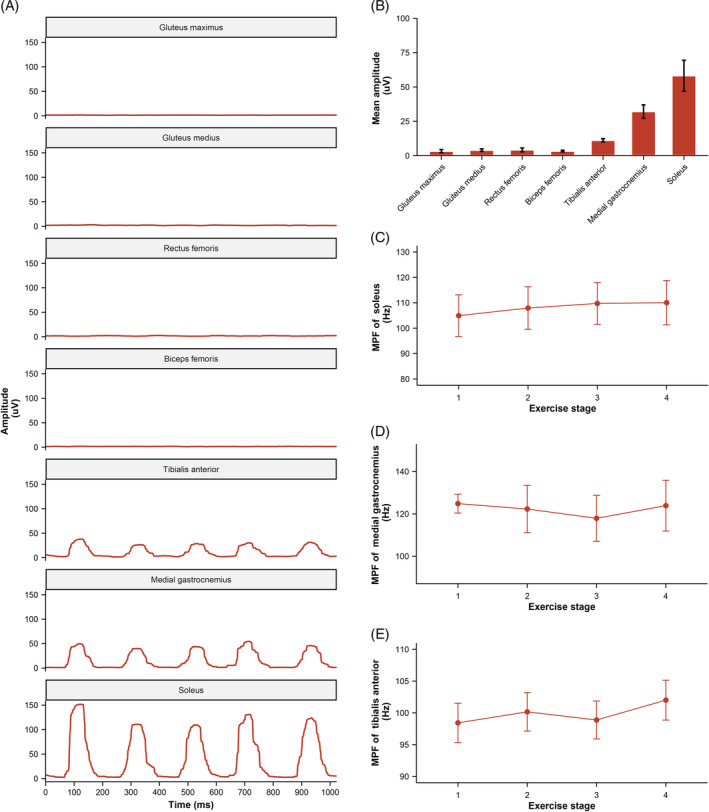
Electromyography (EMG) data during leg shaking. Example of EMG amplitude (A) and mean amplitude (B) during leg shaking. The changes in MPF of soleus (C), medial gastrointestinal (D), and tibialis anterior (E) during leg shaking. Results are in mean ± SEM, and associated *p* values were derived from repeated measures analysis of variance. MPF, median power frequency.

The original EMG signals were filtered, rectified, and smoothed to visualize muscle activation patterns during leg shaking, as illustrated in the left panel of Figure 4. Based on the analysis of the mean amplitude results, it was observed that the soleus, medial gastrocnemius, and tibialis anterior primarily contributed to leg shaking. To further assess muscle fatigue during leg shaking, the MPF of the EMG signals was calculated for these three muscles and no significant changes were observed.

### 3.5 Correlation analysis

**FIGURE 5:**
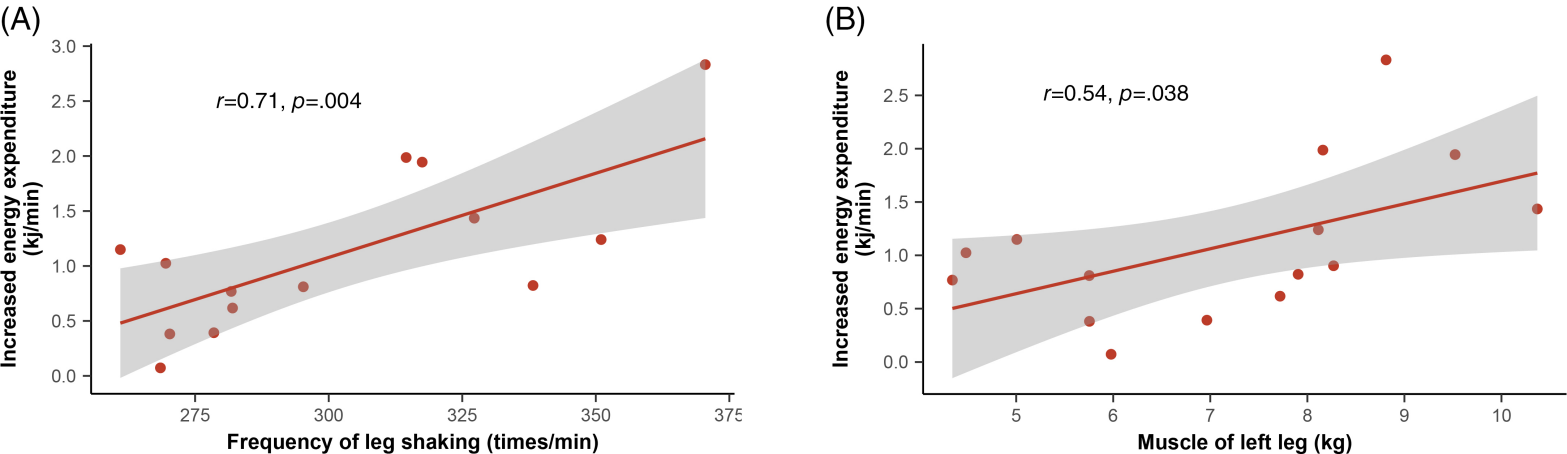
Correlations and 95% confidence limits (dashed area) between frequency of leg shaking (A) and muscle of left leg (B) relative to the increased energy expenditure.

In Figure 5, the correlation coefficient between the increased energy expenditure and the leg shaking frequency was 0.71 (*p* = .004), indicating a strong positive correlation. Similarly, a moderate positive correlation was observed between the increased energy expenditure and the muscle mass of the left leg, with a correlation coefficient of 0.54 (*p* = .038).

## 4 DISCUSSION

The detrimental effects of sedentariness on obesity and chronic diseases are well established.^3^, ^4^, ^5^ However, the prevalence of sedentary behavior continues to increase.^32^ In this study, we investigated the potential of habitual leg shaking as an innovative exercise approach. Our findings revealed that leg shaking significantly increased energy expenditure, primarily contributed by the elevation in carbohydrate oxidation. It also increased respiratory rate, VE, and SpO_2_ levels, while having no obvious impact on heart rate or blood pressure. The analysis of the EMG data confirmed that leg shaking predominantly activated the muscles in lower leg, and there was no evidence of increased muscle fatigue during leg shaking period. Interestingly, we found a significant correlation between the increased energy expenditure and both the frequency of leg shaking and the muscle mass of the leg.

Total energy expenditure (TEE) consists of RMR, diet‐induced thermogenesis, and physical activity‐related energy expenditure (PEE).^33^ PEE exhibits the highest variability and typically accounts for 20%–30% of TEE. In modern society, exercise‐related activity thermogenesis (EAT) often occupies only a negligible portion of PEE, nonexercise‐related activity thermogenesis (NEAT) has emerged as an important factor in body weight regulation.^34^ Our findings demonstrate that leg shaking, as a form of NEAT, led to a significant increase in TEE of ~16.3%. The METs elevated from 1.5 during sitting, which is recognized as an unhealthy SB, to 1.8. Recently, a study investigated the effects of soleus pushup, and the results revealed that this exercise led to approximately a twofold increase in TEE.^35^ Other studies on chair‐based fidgeting has confirmed that using footfidget equipment can significantly increase whole‐body energy expenditure by ~20%–30%.^36^, ^37^ In comparison, the energy expenditure observed in our study was slightly lower. This may be attributed to the fact that we monitored habitual leg shaking, rather than focusing on specific exercise manner or using specific equipment. It is worth noting that despite being the body's largest lean tissue mass, inactive muscles require minimal energy, typically accounting for only 20%–30% of the RMR.^38^, ^39^, ^40^ Researchers found a strong correlation between soleus activation and increased oxygen consumption when sitting.^35^ In our study, the correlation analysis results indicated that both the frequency of muscle activation and muscle mass are crucial determinants of the increased energy expenditure during leg shaking.

Further analysis of substrate oxidation revealed that elevated carbohydrate oxidation played a primary role in the increased energy expenditure during leg shaking. Muscle biopsies of the soleus muscle before and after contraction indicated that muscle glycogen was not the main fuel source for the elevated carbohydrate oxidation but rather free glucose.^35^ Several studies have demonstrated that intermittent leg fidgeting can effectively improve postprandial blood glucose levels and reduce insulin levels.^41^, ^42^ In our study, although the impact of leg shaking on total fat oxidation was relatively negligible, there was a trend of increased fat oxidation during the final stage of shaking. This phenomenon might be attributed to leg shaking duration, as fat oxidation typically rises after ~15 min of fixed‐intensity exercise.^43^ Previous study involving longer durations of muscle contractions also demonstrated a significant enhancement in fat oxidation.^35^ Regarding physiological parameters, we observed a slight increase in SpO_2_, which appeared to be due to a substantial increase in respiratory rate and the corresponding increase in VE.^44^ Pettit‐Mee et al found that intermittent leg fidgeting could significantly improve popliteal artery blood flow.^42^ The enhanced blood flow may also be a potential contributor to the elevation in SpO_2_, as it has been shown that reducing blood flow by elevating the leg can result in a low SpO_2_ reading.^45^ Consistent with previous studies, we did not observe any changes in heart rate and blood pressure.^36^, ^37^ This suggests that leg shaking can effectively increase energy expenditure without adding additional cardiovascular burden, as the intensity of leg shaking may not be sufficient to significantly accelerate heart rate. This low‐effort approach may help avoid the potentially harmful cardiac effects associated with prolonged endurance training in certain individuals or the impairment of mitochondrial function following excessive exercise intensity.^46^, ^47^

EMG analysis confirmed that the activated muscles during leg shaking were the soleus, gastrocnemius, and tibialis anterior in the lower leg, excluding the muscles in the thigh and buttocks. The two most important muscles are the soleus and the gastrocnemius, which are also the largest muscles in the lower leg. They are primarily composed of slow oxidative muscle fibers, accounting for ~70% and 50% respectively.^25^, ^26^ The inherent characteristics make them well‐ suited for long‐duration activity. Previous studies have demonstrated the endurance capacity of these muscles, with participants engaging in intermittent leg fidgeting for up to 3 hours or performing soleus pushup for 4.5 h.^41^, ^42^, ^48^ The participants responded well to the prolonged muscle contractions and did not experience fatigue or other adverse responses.^35^ To assess muscle fatigue, the shift in MPF is a well‐established method.^49^ MPF analysis showed no increasing fatigue trend in the three major muscles involved during leg shaking. This suggests that leg shaking can be performed continuously during prolonged SB.

It is important to note that our study has several limitations. First, the sample size was relatively small, and the participants were primarily healthy young adults, which may limit the generalizability of the findings. Second, although the participants simulate habitual leg shaking, the entire study was conducted in a laboratory setting. Further investigation is needed to explore leg shaking in a real‐world setting. Additionally, the duration of leg shaking in our study was relatively short. Long‐term studies investigating the sustained effects of leg shaking on SB and health outcomes are warranted.

In conclusion, leg shaking is usually considered as a common negative repetitive behavior. In China, there is even an old saying, “Men shake to poverty, women shake to cheap,” which reflects the negative perception toward leg shaking. However, it is crucial to challenge and change this traditional perception. Our study confirmed that habitual leg shaking effectively increased energy expenditure, elevated the metabolic equivalent to a nonhealthy level, enhanced carbohydrate oxidation, improved SpO_2_ and VE, while avoiding any additional cardiovascular burden. It offers a simple and feasible approach that could be easily performed anywhere and at any time, without disturbing daily work and life routines. Due to the slow oxidative muscle characteristics, prolonged and continuous leg shaking becomes feasible. Assuming 8–12 h of continuous leg shaking, it could result in 524.42–786.63 kj of additional energy expenditure. By promoting physical activity in sedentary individuals, leg shaking has the potential to contribute to public health initiatives aimed at reducing the burden of obesity and related chronic diseases.

## AUTHOR CONTRIBUTIONS

Weiqing Qang and Shijia Pan conceived and designed the trial. Riqiang Bao, Yixiang Hu contributed to the protocol and design of the study. Riqiang Bao, Chong Gao, Rui Xu contributed to the implementation of the study. Yixiang Hu, Yuhan GUo and Yashu Zhu contributed to the data collection. Riqiang Bao contributed to the manuscript preparation. All authors have read and approved the final version of the manuscript and agree with the order of presentation of the authors.

## FUNDING INFORMATION

This study was sponsored by the grants from the National Key Research and Development Program of China (2021YFC2501600, 2021YFC2501603) and the Innovative Research Team of High‐Level Local Universities in Shanghai (91857205). The funding bodies had no role in the study design or in data collection, analysis, or interpretation.

## CONFLICT OF INTEREST STATEMENT

The authors have nothing to disclose.

## Data Availability

The datasets used and/or analyzed during the current study are available from the corresponding author on reasonable request.
